# The Air We Breathe

**Published:** 1870-01

**Authors:** 


### THE AIR WE BREATHE.

In ordinary breathing, a man takes into his lungs, at one inspiration, about a pint of air. It enters pure, and is returned so destitute of life, that if it were immediately rebreathed, without the admixture of other air, the man would die on the spot. This outbreathed air is loaded with the impurities and wastes of the system, which have been reduced to a vapory and gaseous state, and carried to the lungs in the blood, where an exchange takes place; the pure air imparts its life to the blood, and the blood loads it with its death, to be freighted out of the system, which is thus kept in healthful working order.

But if the air taken into the lungs is not pure, it fails to take the proper proportion of impurities out of the system; hence these impurities increase, rendering the blood more and more impure every day; making it thicker and thicker, until it is almost too thick and black to move at all; and in this state it clogs up, accumulates, causing ailments, named from the parts affected. If this clogging up takes place in the brain, it causes apoplexy; if in the bowels, it is called dysentery, or bloody flux; if in the lungs, it causes congestion, or pneumonia, or lung fever; these three dangerous maladies being essentially one and the same.

The importance, then, of being out of doors every day as much as possible, can scarcely be overrated. But to derive the highest advantage from breathing an out-door air, the person should exercise enough to keep off a feeling of chilliness, but not so much as to cause unnatural breathing —that is, too fast or too deep.

